# Assessing the Sulfide Footprint of Mussel Farms with Sediment Profile Imagery: A New Zealand Trial

**DOI:** 10.1371/journal.pone.0129894

**Published:** 2015-06-17

**Authors:** Peter S. Wilson, Kay Vopel

**Affiliations:** Institute for Applied Ecology New Zealand, School of Applied Sciences, Auckland University of Technology, Auckland, New Zealand; Institut Maurice Lamontagne, CANADA

## Abstract

Growing numbers and increased stocking of marine mussel farms make reliable techniques for environmental effect assessment a priority. Previously, we showed how the color intensity of soft sediment could be used to estimate its acid volatile sulfide (AVS) content, a product of the anaerobic microbial degradation of organic matter deposits. We then proposed to include assessments of the AVS farm footprint in marine farm monitoring, in particular, to investigate temporal changes in the extent of the seafloor area of elevated sediment AVS content. Such assessment requires accurate detection of the AVS footprint boundary. Here, we demonstrate how to detect this boundary with analyses of sediment color intensity. We analyzed 182 sediment profile images taken along three transects leading from approximately 50 m inside to 200 m outside a long-line mussel farm in New Zealand and found that the mean sediment color intensity inside the farm boundary was almost one third lower than that of the sediment distant from the farm. Segmented regression analysis of the combined color intensity data revealed a breakpoint in the trend of increasing grey values with increasing distance from the farm at 56 ± 13 m (± 95% confidence interval of the breakpoint) outside the mussel farm. Statistical analyses indicated that the extent of the color intensity footprint was a function of water column depth, as was shown visually using mapping methods; organic particles disperse further in a deeper seawater column. We conclude that for soft coastal sediments, our sampling and data analysis techniques may provide a rapid and reliable supplement to existing benthic surveys that assess environmental effects of mussel farms.

## Introduction

Global food production from marine farms has increased on average 6.2% per year since 2000, rising to 90.4 million tons in 2012 [1, 2]. Of all marine-cultured species, bivalves contributed over 15 million tons. The introduction of new farms to the coast, and increased stocking of existing farms necessitates the development of rapid and reliable techniques for the assessment of the effects that such farms have on coastal ecosystems.

Suspended bivalve farms can alter their ecosystems to various degrees depending on the farm’s size, age, and stocking density, the seawater column depth and flow regimes, season, and climatic conditions [3–6]. Ecosystem effects may arise from mussel feeding habits, farm structures, and activities associated with mussel cultivation and harvest. Documented effects include changes in local hydrodynamics [7], phytoplankton depletion [8, 9], the spread of invasive organisms [10], and the deposition of farm-derived organic matter (mussel feces and pseudofeces, [11]). The latter can increase the sulfide and ammonium content of the sediment below mussel farms altering the structure and composition of benthic species assemblages [4, 12, 13]. Mattsson and Lindén [3], for example, reported that the dominant heart urchin and brittle star had been replaced by opportunistic polychaetes 15 months after the introduction of a suspended mussel farm in ~15 m deep water of a sheltered bay on the Swedish west coast. Other effects may result from the provision of additional hard substrate due to dropping shells: aggregation of sessile suspension feeder including ascidians, bryozoans, sponges, bivalves, and calcareous polychaete species. Such alterations increase the surface roughness and heterogeneity of the seafloor and create a reef-like habitat for a variety of mobile species including fish, crustaceans, and various echinoderms [13, 14].

These and other alterations of the benthic environment are horizontally confined to an area beneath and possibly around the farm, which hereafter we refer to as the “footprint”. In New Zealand, for the purpose of marine farm monitoring, environmental managers ask if and how this footprint changes over time. Once a new farm is fully operational its footprint may not change over time if the interaction of this farm with its surrounding environment reached a steady state. Alternatively, the extent and/or the intensity of the footprint may increase over time. To identify, and if existing, quantify such increase, marine farm monitoring should assess two variables: (1) the size of the affected seafloor area, and (2) the degree to which the affected seafloor differs from the unaffected seafloor. Various approaches have been used worldwide to describe these variables, for example: detecting mussel debris with side scan sonar [15] or sediment grab samples [14], identifying genetic differences in sediment microbial communities [16], modeling biodeposit dispersion [17], and measuring the total free sulfide content of the sediment, the sediment redox potential, and water and organic matter contents [18].

In recent years, environmental scientists have attempted to assess the footprint of mussel [19] and fish farms [20–22] by measuring the depth of the apparent redox potential discontinuity (aRPD) with sediment profile imagery (SPI). SPI analyses can be supplemented with that of sediment surface images [23] and other visual indicators, such as the presence or absence of fauna and their burrows, in addition to the depth of the aRPD. A combination of these parameters can be included in analyses to calculate benthic indices, for example, the organism–sediment index (OSI, [24, 25]), the benthic habitat quality index (BHQ, [26, 27]), and the Galway Bay index of habitat quality (GBHQ, [28]). The latter index is site-specific allowing greater differentiation of intermediate environmental states along a local enrichment gradient (see also [29]).

Bull and Williamson [30] used sediment profile image analysis to quantify sedimentary minerals based on a relationship between a specific sediment color property and the mineral concentration. The authors reported two linear correlations for the subtidal sediment of a New Zealand estuary: one between sediment color intensity and acid volatile sulfide (AVS) concentration (*R* ^2^ = 0.67), and the other between sediment color saturation and iron oxyhydroxide (FeOOH) concentration (*R* ^2^ = 0.62). Sediment color intensity and AVS concentration are inversely related, such that a decrease in sediment color intensity (darker sediment) accompanies an increase in AVS concentration. The authors established each correlation by slicing sediment cores vertically and photographing the exposed surface. They then analyzed subsamples of the sediment for AVS and FeOOH content, which were correlated with the corresponding color property.

The correlation between sediment color intensity and AVS concentration is of particular interest for assessing the environmental effect of marine farms because the sediment AVS concentration is a function of the organic matter deposition rate [12, 31–37]. To the best of our knowledge, however, AVS measurements are not used in routine monitoring of marine farms presumably because of the laborious analytical process. Estimating the AVS concentration from sediment profile images is rapid [38], however, and could make AVS surveys a valuable supplement to existing benthic monitoring techniques.

Wilson and Vopel [38] further developed the approach of Bull and Williamson [30] and used their improved technique to establish a site-specific correlation between sediment color intensity and AVS concentration. They studied soft subtidal sediment affected by a long-line mussel farm that had been operating since 1980 in Awakiriapa Bay, Waiheke Island, New Zealand. The authors reported a strong quadratic relationship between sediment color intensity and AVS concentration (*R* ^2^ = 0.93) and suggested to use this correlation in the monitoring of long-line mussel farms, that is, to investigate temporal changes in (a) the color intensity of sediment underneath such farms and (b) the extent of the footprint, that is, the seafloor area of decreased sediment color intensity (elevated sediment AVS content). The latter requires an approach to accurately detect the location of the footprint’s boundary.

**Fig 1:**
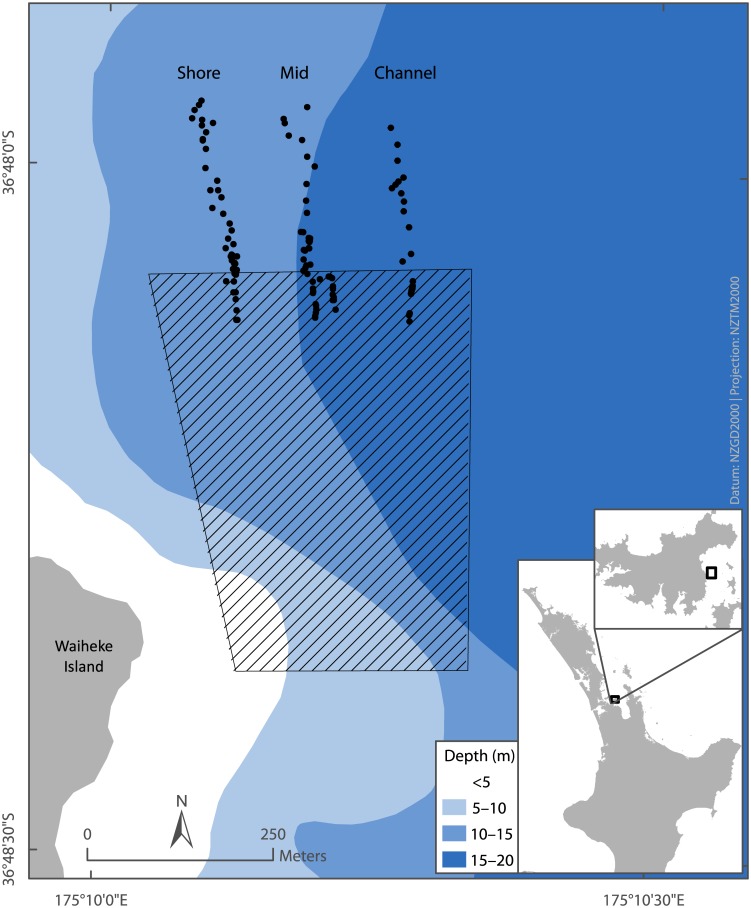
Map showing the location of the long-line mussel farm in Awakiriapa Bay, Waiheke Island, New Zealand. The hashed area indicates the long-line mussel farm. Each black symbol represents one sediment profile image taken along one of three transects.

Here, we demonstrate such approach with a series of sediment profile images obtained along transects leading from inside to outside of a long-line mussel farm in Awakiriapa Bay, Waiheke Island, New Zealand (Fig 1).

## Methods

### *In situ* SPI survey

We acquired 182 profile images of soft subtidal sediment during three days in April 2013 and one day in June 2013 along three transects at the Awakiriapa Bay long-line mussel farm, Waiheke Island, New Zealand (S36°48.085', E175°10.022'; Fig 1). Each transect started approximately 50 m inside the farm boundary and extended north, ~200 m beyond the farm boundary. The farm boundary is defined as the position of the end buoy of the mussel line. We didn’t require any specific permission to obtain images from this site as it was in public waters and there were no endangered or protected species.

We acquired sediment profile images using SPI-Scan, a rotational imaging device (Benthic Science Ltd., see Wilson and Vopel [38]). The sediment penetration depth of the instrument was adjusted using two approaches, (1) attaching 3–8 × 1 kg weights to the top of the device and (2) controlling the speed at which the instrument penetrated the sediment by either releasing the instrument approximately 2–3 m from the seafloor, or slowly lowering the instrument to the seafloor. We made such adjustments so that the sediment filled two-thirds of the image. Sediment profile scanning was started immediately after the device was in place.

**Fig 2:**
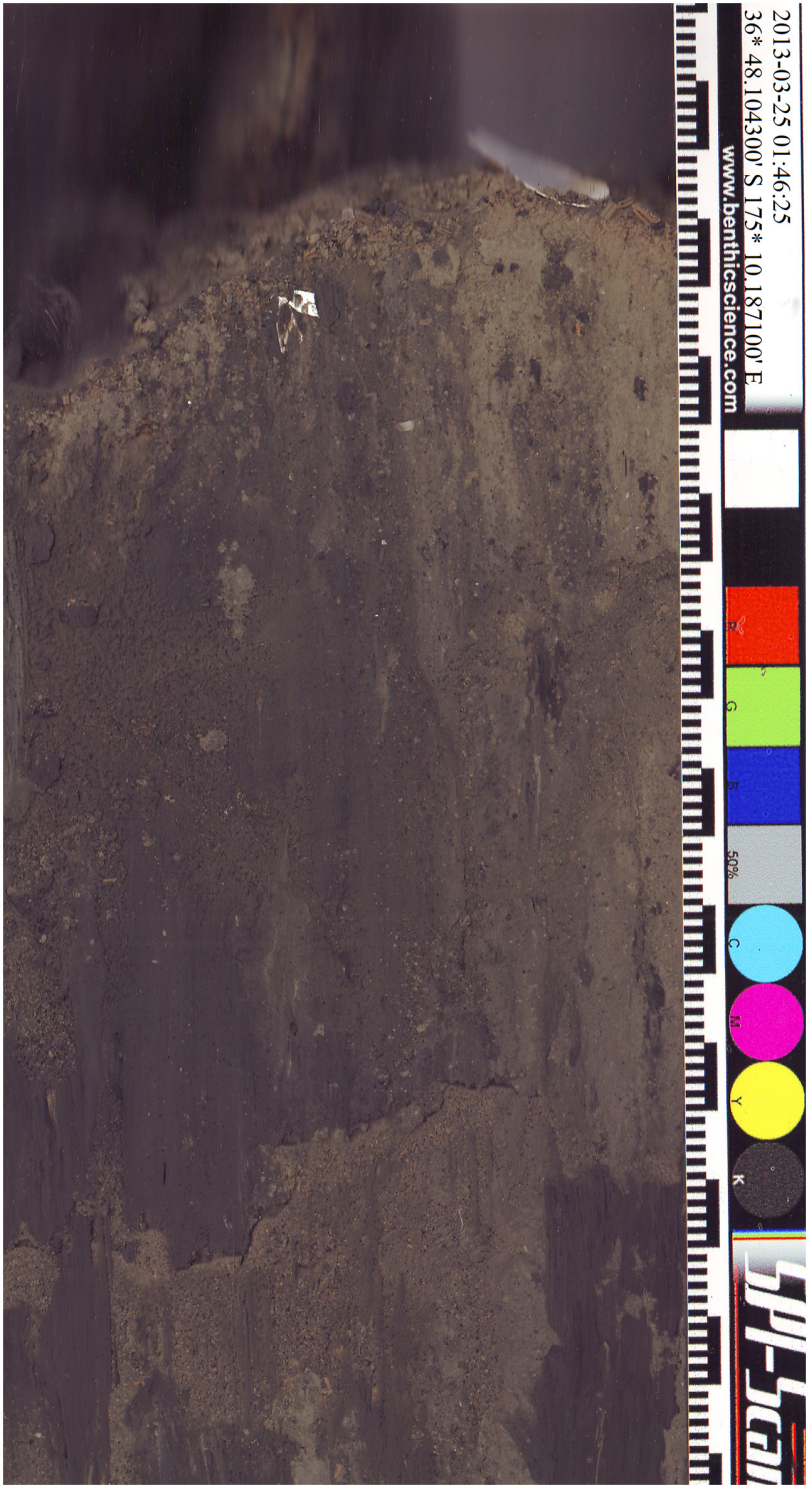
Example *in situ* sediment profile image obtained with SPI-Scan. The small and large black and white bars on the right hand side indicate 1 and 10 mm. Color references are used for color calibration of the digital image.

The software (SPI-Scan) digitally embedded the GPS coordinates, date, and time of the acquired image in the metadata of each image. This information is also presented in the upper right-hand corner of the image (Fig 2).

**Fig 3:**
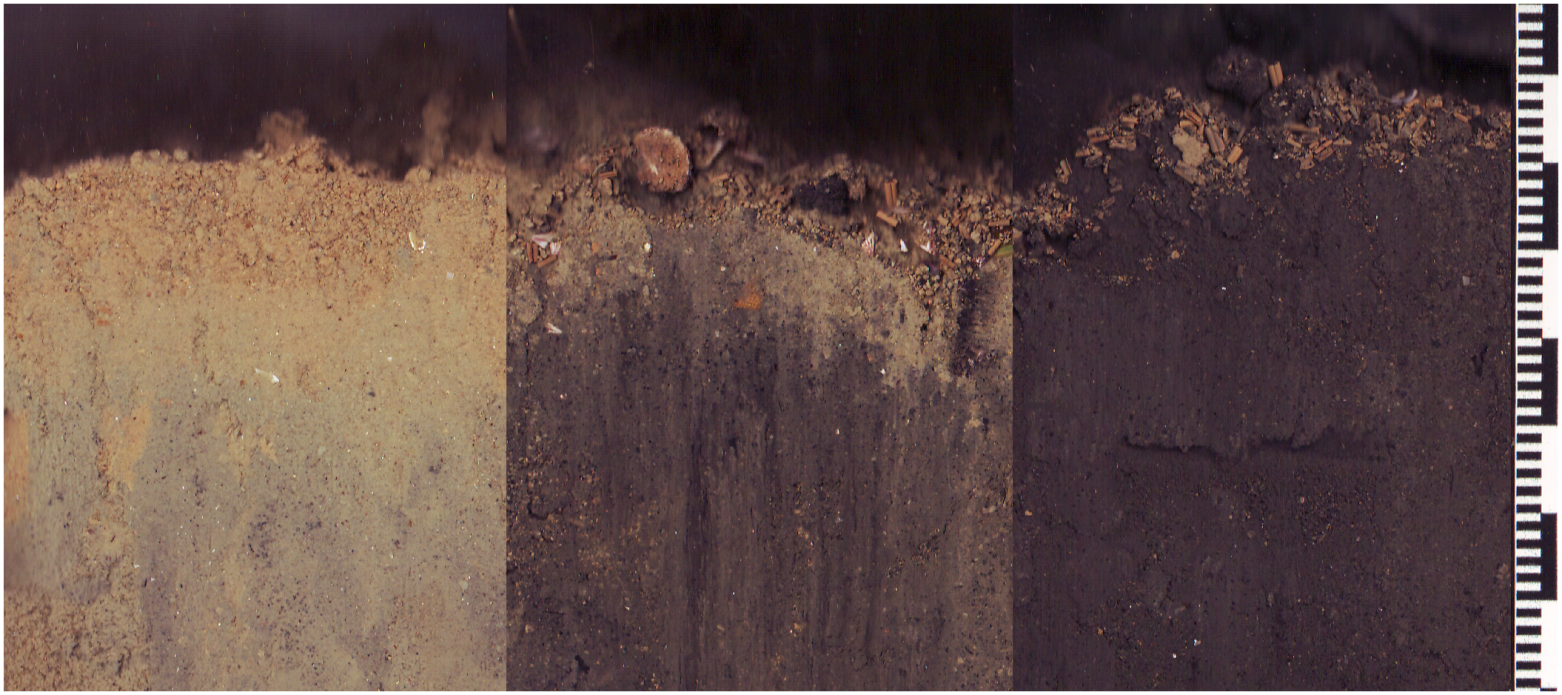
Example sediment profile images showing the range of sediment colors in Awakiriapa Bay, Waiheke Island, New Zealand. The left image was obtained 48 m north of the northern farm boundary, and the middle image and right images 35 and 57 m south of the northern farm boundary. The small and large black bars are 1 and 10 mm.

A color calibration strip was included on the right hand side of each profile-image. We used the calibration strip during image analysis to adjust the color of the image so that the color reproduction of each image was identical. Each profile image covered an area of 117 × 216 mm at a resolution of 300 dpi (0.08 mm pixel^-1^). Each scan took approximately 30 seconds to complete; this excludes the time required to lower the instrument from the boat or move the instrument, while underwater, to a new location. An example of the color-range of imaged sediments is shown in Fig 3.

### SPI analyses

**Fig 4:**
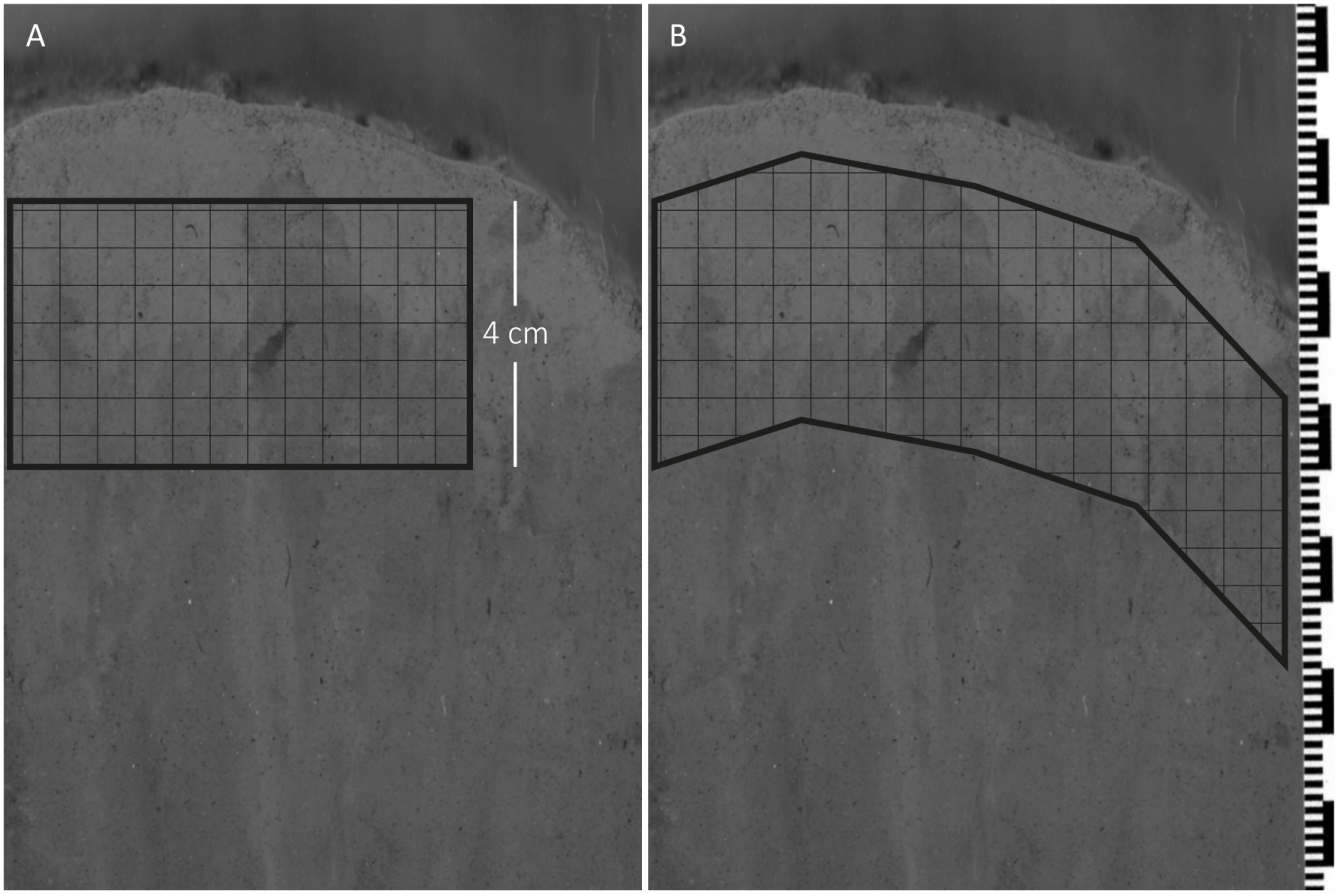
Two methods for selecting the area of interest for image analysis. The grey-scale images were derived from a full color image by extracting the color intensity channel in the HSI color space. A, a rectangle starting approximately 1 cm from the sediment surface; B, a polygon that follows the contour of the sediment surface, starting approximately 1 cm below the sediment surface. The small and large black bars are 1 and 10 mm.

To measure the color intensity (grey value) of each sediment profile image, we first imported all images into the software analySIS FIVE (Olympus Soft Imaging Solutions, LS Research version 3.3). We then converted the color image from the red, green, blue (RGB) color space to the hue, saturation, intensity (HSI) color space and measured the average grey value of the intensity channel of a defined area in the sediment profile image, as described below. We observed during image analysis that the sediment surface in the profile images was rarely horizontal and therefore trialed two different methods for selecting an area for image analysis, (1) a rectangle starting approximately 1 cm below the sediment surface that extended 4 cm down, and (2) a polygon that followed the contour of the sediment surface, starting 1 cm below the sediment surface and extending 4 cm down (Fig 4).

Because the mean grey values of the rectangular image area did not differ from those of the polygonal area (paired-samples t-test, *t*(174) = 0.49, *p* = 0.62), both methods would have sufficed. In this study, however, we used the larger polygon (~400,000 versus ~260,000 pixels^2^) because the selection of this area was less ambiguous than that of a rectangular area.

Finally, we converted measured sediment color intensities to sediment AVS concentrations using the equation published by Wilson and Vopel [38] for the Awakiriapa Bay long-line mussel farm: [AVS] = 0.0024 × GREY^2^ − 0.5249 × GREY + 28.392. Please note that not all measured grey values could be accurately converted to AVS concentrations because some were outside the range of grey values used by Wilson and Vopel [38] to establish this equation.

### Statistical analyses

We investigated whether there was any statistically significant difference between transects by analyzing grey values inside the farm (distance from farm boundary <0 m), outside of the farm (distance from farm boundary >0 m), and overall separately with ANCOVA. Where there was a significant difference, we additionally explored the effect of seawater depth, transect, and distance from the farm boundary on the grey value with a full factorial ANCOVA.

To identify the boundary of the color intensity footprint, we performed a segmented regression analysis with the ‘segmented’ package in R [39, 40]. We performed this analysis for each of the three transects and on a dataset created by combining the data from all three transects. The analysis used an iterative procedure to fit two linear regressions and find a breakpoint in the data trend by minimizing the sum of squares of the differences between observed and calculated variables. We constrained the slope of the second linear regression to zero. We did so because we were trying to identify a color change from the unaffected sediment surrounding the farm (background). We assumed that although the color intensity of this sediment will naturally vary, there would be no significant long-distance trend of increasing or decreasing color intensity. We ran the segmented model using distance from the farm boundary as the predictor and a starting (psi) value of 100. We varied psi from 50 to 150 to ensure this value was not biasing the result.

### Color intensity mapping

We used marine farm location data (Land Information New Zealand) in ArcMap (ESRI ArcGIS, version 10.2) to determine the distance of each sampling point from the northern boundary of the mussel farm. To do so, we imported the GPS coordinates embedded in each sediment profile image and then calculated the distance from the image location to the northern edge of the mussel farm with the Generate Near Table tool in ArcMap. The depth information for each data point was extracted from a 20 m resolution gridded bathymetric dataset [41]. We mapped the sediment color intensity over an area extending from ~50 m inside the northern farm boundary to ~200 m north of the farm boundary with the polynomial interpolation model in ArcMap (see S1 Table for model parameters). The model extrapolated the measured sediment color intensities to cover this area.

## Results

Sediment color intensities (grey values) were high and varied little (93 ± 1 grey values; mean ± 95% CI, *n* = 83) at distances >50 m north of the northern farm boundary. This defines the “background” sediment color intensity in Awakiriapa Bay. The mean sediment color intensity inside the farm, that is, south of the northern farm boundary, was almost one third lower (61 grey values) than the background color intensity. This lower sediment color intensity defines the footprint of the farm.

**Fig 5:**
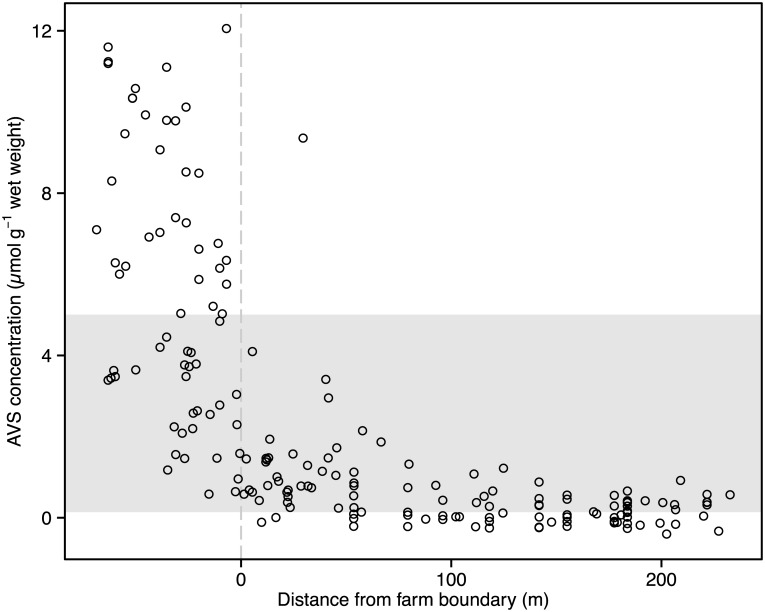
Sediment AVS concentrations along three transects crossing the northern boundary of a mussel farm in Awakiriapa Bay, Waiheke Island, New Zealand. AVS concentrations were predicted from measured sediment color intensities using the correlation equation in Wilson and Vopel [38]. AVS concentrations inside the grey area were derived from grey values within the range of grey values presented by Wilson and Vopel [38] in their AVS/color intensity correlation; AVS concentrations outside of this grey area were extrapolated using the correlation equation.

Conversion of the measured sediment color intensities to AVS concentrations with the AVS/color intensity correlation published by Wilson and Vopel [38] revealed AVS concentrations far above the range of those in the published correlation for nearly half of all profile images inside the farm, and negative concentrations for some profile images of sediment outside the farm (Fig 5). The latter concentrations were all within the margin of error of the AVS/color calibrations if the actual AVS concentrations were at or near 0 μmol g^−1^; the mean 95% individual CI of the AVS/color calibration was 0.5 μmol g^-1^ [38].

**Fig 6:**
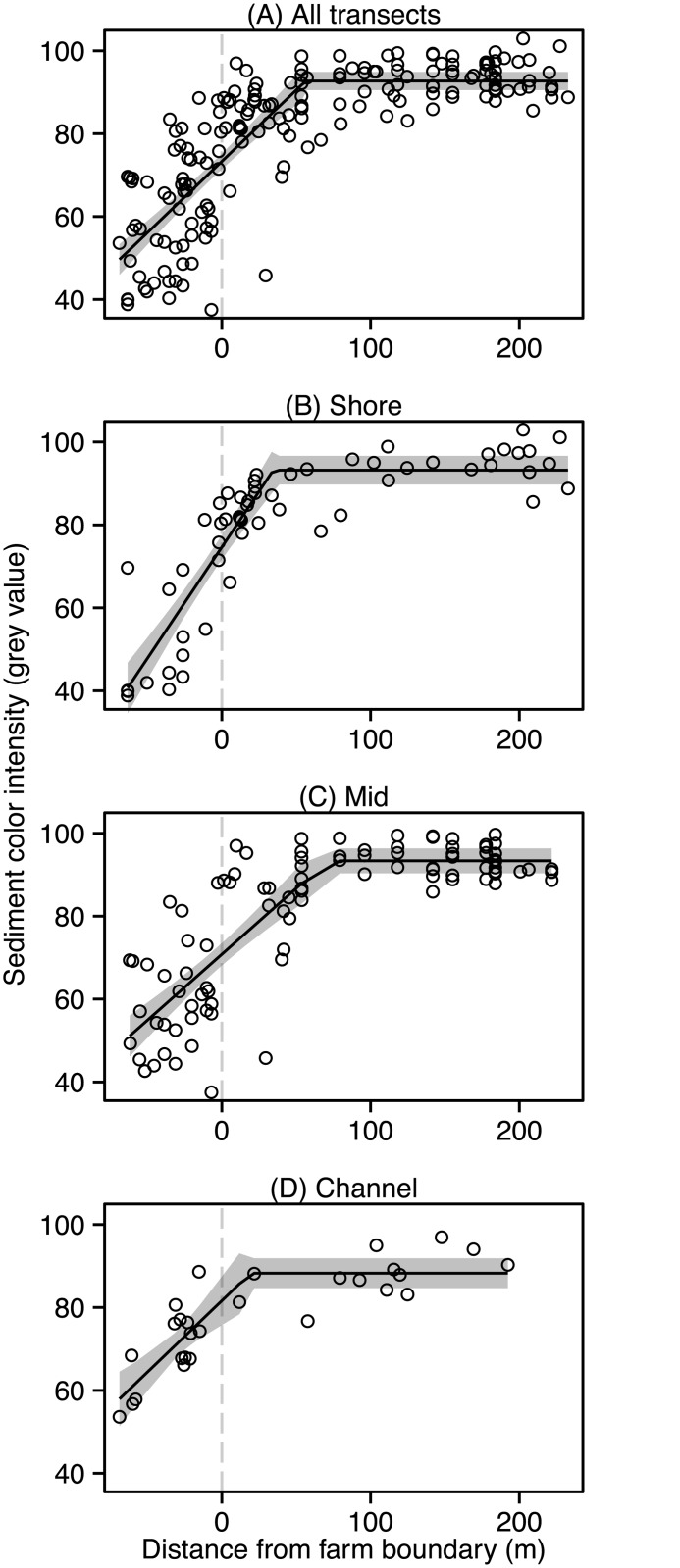
Segmented regression analysis. A segmented regression model identified the distance from the northern farm boundary at which the color intensities of the sediment in Awakiriapa Bay, Waiheke Island, New Zealand started to decrease when heading towards the farm. The analysis was performed on data combined from the three transects (A) and separately for each transect (B–D). The grey shaded area shows the 95% confidence interval of the model and the vertical grey dashed line denotes the mussel farm boundary. Negative distances indicate points within the farm, that is, south of the northern farm boundary.

In the following we locate the position of the AVS footprint boundary using color intensity data instead of AVS data to avoid uncertainty from extrapolating the calibration in Wilson and Vopel [38]. Segmented regression analysis of the color intensity data from all transects located the northern boundary of the footprint at 56 ± 13 m (± 95% confidence interval of the breakpoint) outside and north of the mussel farm (Fig 6A). Furthermore, segmented regression analysis on individual transects revealed that the footprint extended 35 m less on the shore side than it did in the middle of the farm (Fig 6B–6D, Table 1).

**Table 1:** Extent of the Awakiriapa Bay mussel farm color intensity footprint.

Transect	Footprint extent (m)	95% CI	*R* ^2^	*N*
All	56	13	0.69	182
Shore	35	11	0.81	57
Mid	71	21	0.70	97
Channel	19	27	0.76	28

The extent of the mussel farm color intensity footprint was determined by segmented regression analysis for each transect individually, and for the combined set of sediment color intensity data.

The mean sediment color intensity inside the farm was lower (darker sediment) along the Shore and Mid transects than it was along the Channel transect (Table 2).

**Table 2:** ANCOVA used to investigate differences in the color intensity of sediment along three transects across the Awakiriapa Bay mussel farm boundary, Waiheke Island, New Zealand.

	Df	Residuals	*F*	*p*	*R* ^2^	Tukey contrast
Overall	2	178	1.13	0.324	0.56	
Inside farm	2	61	6.84	**0.002**	0.33	Shore, Mid < Channel
Outside farm	2	113	0.57	0.558	0.25	

Overall includes all data points for each transect, Inside farm and Outside farm include data points for distances <0 and >0 m from the northern farm boundary. Where the result was significant (*p* > 0.05), we used a Tukey post-hoc contrast to determine ranking. The model equation was: Grey = Distance + Transect.

To elucidate the intensity increase of the footprint towards the shore side of the farm, we investigated how the variables Depth, Distance from the farm boundary, and Transect influenced sediment color intensities inside the mussel farm and found that all three variables, and some interactions between these variables, had a significant effect (Table 3). The Shore transect was closest to the shore and in seawater two meters shallower than that of the other two transects (Table 4).

**Table 3:** Full factorial ANCOVA used to investigate the effect of Distance from the farm boundary, Transect, and Depth on the color intensity of the sediment inside the farm (distance <0 m from the farm boundary).

Inside (*R* ^2^ = 0.56)	Df	Sum Sq	Mean Sq	*F*	*p*
Distance	1	2274.9	2274.9	22.3	**<0.001**
Transect	2	1849.1	924.5	9.1	**<0.001**
Depth	1	442.7	442.7	4.3	**0.042**
Distance:Transect	2	639.6	319.8	3.1	0.051
Distance:Depth	1	89.8	89.8	0.9	0.352
Transect:Depth	2	973.2	486.6	4.8	**0.012**
Distance:Transect:Depth	2	701.7	350.9	3.4	**0.039**
Residuals	53	5398.4	101.9		

**Table 4:** Mean water depth and mean sediment color intensity for each of three transects within the mussel farm.

	Depth (m)	95% CI	Color intensity (grey value)	95% CI	*N*
Shore	13.6	0.04	58	8	18
Mid	15.3	0.03	59	5	32
Channel	15.7	0.003	70	5	15

The final column indicates the number of samples that were averaged.

**Fig 7:**
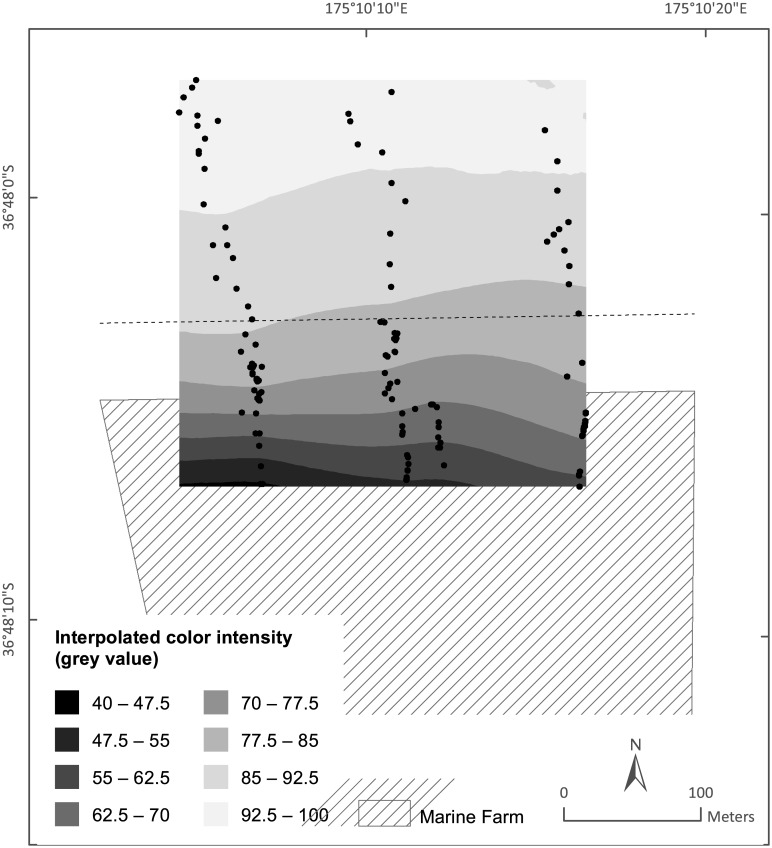
Sediment color intensity footprint of the Awakiriapa Bay mussel farm, Waiheke Island, New Zealand. A local polynomial interpolation model (ArcMap) extrapolated the color intensity of the sediment imaged along three transects (black symbols) to map the sediment color intensity in the shaded area. Sediment color intensity is correlated to AVS concentration [30, 38]. The hashed area denotes the mussel farm. The dashed line 56 m north of the northern farm boundary denotes the edge of the mussel farm AVS footprint, as identified from segmented regression analysis of sediment color intensity data.

The color intensity footprint map in Fig 7 supports the results of our segmented regression analysis: sediment color intensities were >80 grey values at distances greater than 50 m from the farm boundary and <77.5 grey values at distances less than 50 m from the farm boundary. This map also indicated that the color intensity footprint didn’t extend as far on the western side of the farm as it did on the eastern side.

## Discussion

We demonstrated the use of *in situ* SPI to assess the sediment color intensity footprint of a long-line mussel farm. Two variables were of interest, the intensity of the footprint, that is, the magnitude of the difference between the color intensity of the sediment underneath the farm and that of the surrounding unaffected sediment, and the size or spatial extent of the footprint. For our trial mussel farm, we determined the latter with segmented regression analysis of our color intensity data (Fig 6, Table 1); the footprint extended approximately 50 m beyond the northern boundary of the mussel farm. This distance lies within the range of numerical model predictions of the dispersion and erosion of feces and pseudofeces at a nearby mussel farm, 25 km southeast of Awakiriapa Bay. Here, Giles et al. [17] found that mussel feces released by this farm into a 10 m deep seawater column were deposited up to 60 m away from the mussel farm boundary. Incorporating resuspension by erosion into their model increased this distance to 130 m. Clearly, the deposition of small amounts of feces and pseudofeces away from the farm does not necessarily result in a detectible decrease of sediment color intensity, but such deposition modeling can inform the design of future AVS and color intensity footprint surveys. In particular, it will help to ensure that survey transects extend far enough from the mussel farm boundary to include the unaffected seafloor.

Two advantages of this SPI analysis technique are that it is rapid and it does not require in-depth training and experience of the operator to measure and adequately interpret the ecological significance of image parameters. The operator simply uses software routines to measure one property of the sediment, its color intensity. Selecting an image area to determine this property is the only step in our routine that requires the operator to make a decision. We believe that its simplicity and automation make this method suitable to complement other types of assessments of the effects of organic enrichment of sediments underneath mussel or fish farms on the soft-sediment ecosystem because it will ensure that data acquisition remains consistent over the lifetime of a monitoring program. The placement of color intensity survey transects at the onset of such program must consider local differences in seawater depth and current speed—two variables that affect the dilution and dispersion of feces and pseudofeces after release [42, 43].

For our trial mussel farm, differences in the sediment color intensities between the three transects and the sediment color intensity map in Fig 7 indicate that the farm AVS footprint did not extend as far beyond the farm boundary close to the shore as it did close to the channel, logically because the seafloor underneath the shore side farm blocks is less deep and thus biodeposits would not disperse as far. Hartstein and Stevens [44] support this reasoning; their sediment trap measurements and dispersal model revealed that because particles released from a farm in shallower seawater spend less time in the water column they deposit over a smaller area of seafloor than particles released in deeper seawater. Consequently, deposition in shallow seawater will elevate the sediment organic matter content more than deposition in deeper seawater. Differences in water depth cannot explain the differences in the sediment mean color intensity between the Mid and Channel transects within the farm as their depths did not differ significantly. We suggest that this difference resulted from a higher flow speed along the Channel transect, which has a similar effect as a greater water depth, increasing the dispersion of suspended particles from the mussel farm.

Over half of the sediment color intensities measured inside the mussel farm were outside the range of those color intensities included in the AVS/color intensity correlation presented by Wilson and Vopel [38]. Clearly, a new calibration covering a wider range of sediment color intensities is required to accurately predict the entire range of AVS concentrations and any conclusions made from the extrapolated AVS concentrations should be conservative. Many of the AVS concentrations derived for the sediment in the area between the farm boundary and the footprint boundary, however, are within the calibrated range and so suitable for comparison with future measurements. Such measurements outside the farm boundary may reveal that the sediment color intensities have decreased over time and this will indicate that the intensity and extent of the AVS footprint of this farm has increased. Environmental managers may use such trend to request more detailed investigations of the effect of organic matter deposition on benthic ecosystem functioning.

We mapped sediment color intensities to visually represent the two-dimensional color intensity footprint. Such representation may simplify any assessment of the shape of the footprint but it cannot provide any more detailed information than the three transect measurements from which it was derived. The availability of resources for mussel farm monitoring often limit the number of sites, measured parameters, and the frequency at which measurements are conducted. If so, we recommend focusing data acquisition to produce sufficient sediment profile images along one transect rather than aiming for greater spatial coverage. The minimum number of scans will depend on local conditions and this should be investigated with a trial before commencing monitoring. In this study, segmented regression identified the footprint boundary along the Channel transect with as few as 28 data points. Segmented regression analyses of, for example, annual data sets will then allow investigators to assess if the position of the footprint boundary in the direction of this transect or the intensity of the footprint, that is, the color intensity of the sediment underneath the farm change over time. If the shape of the footprint and local differences in its extent are of interest, however, the sampling design should ensure sufficient spatial coverage to produce color intensity maps as shown in Fig 7.

Deposition of farm-derived organic matter alters both the redox chemistry of the receiving soft-sediment seafloor and the composition of its benthic species assemblages [45–47]. Changes in these two variables are linked; soft sediment with elevated organic matter often supports assemblages dominated by few opportunistic species. The seafloor underneath mussel farms, however, accumulates hard substrate in form of dropped mussel shells and this accumulation can lead to contrasting results. In a previous study of our trial mussel farm in Awakiriapa Bay, for example, Wong and O'Shea [14] demonstrated that species richness and diversity of the macrofaunal assemblage were higher beneath than outside the mussel farm. The authors suggested that the boundary of the farm footprint (based on attributes of the benthic macrofaunal assemblage) was marked by the outer limit of benthic clumps of mussel shells, which extended 30 m past the boundary of the mussel farm. Here, instead of using clumps of dropped mussel shells as an indicator, we used a property of the soft-sediment ecosystem that is linked with the deposition of farm-derived organic matter and demonstrated that the footprint of this farm extended beyond 30 m.

## Supporting Information

S1 Table Parameters for the local polynomial interpolation model in ArcMap (ESRI ArcGIS, version 10.2). Measured sediment color intensities along three transects running across the boundary of the mussel farm in Awakiriapa Bay, Waiheke Island, New Zealand were extrapolated over an area extending from ~50 m inside the northern fam boundary to ~200 m north of the farm boundary.
(DOCX)

## References

[1] FAO. The state of world fisheries and aquaculture. Rome: Food and Agriculture Organization of the United Nations, 2014.

[2] Godfray HCJ, Beddington JR, Crute IR, Haddad L, Lawrence D, Muir JF, et al Food Security: The Challenge of Feeding 9 Billion People. Science. 2010;327(5967):812–8. 10.1126/science.1185383 20110467

[3] Mattsson J, Lindén O. Benthic macrofauna succession under mussels, *Mytilus edulis* L. (Bivalvia), cultured on hanging long-lines. Sarsia. 1983;68(2):97–102. 10.1080/00364827.1983.10420561

[4] Grant J, Hatcher A, Scott DB, Pocklington P, Schafer CT, Winters GV. A multidisciplinary approach to evaluating impacts of shellfish aquaculture on benthic communities. Estuaries. 1995;18(1 A):124–44. 10.2307/1352288

[5] Crawford CM, Macleod CKA, Mitchell IM. Effects of shellfish farming on the benthic environment. Aquaculture. 2003;224(1–4):117–40. 10.1016/S0044-8486(03)00210-2

[6] Hartstein ND, Rowden AA. Effect of biodeposits from mussel culture on macroinvertebrate assemblages at sites of different hydrodynamic regime. Mar Environ Res. 2004;57(5):339–57. 10.1016/j.marenvres.2003.11.003 14967518

[7] Plew DR, Stevens CL, Spigel RH, Hartstein ND. Hydrodynamic implications of large offshore mussel farms. IEEE J Ocean Eng. 2005;30(1):95–108. 10.1109/JOE.2004.841387

[8] Grant J, Bacher C, Cranford PJ, Guyondet T, Carreau M. A spatially explicit ecosystem model of seston depletion in dense mussel culture. J Mar Syst. 2008;73(1–2):155–68. 10.1016/j.jmarsys.2007.10.007

[9] Duarte P, Labarta U, Fernández-Reiriz MJ. Modelling local food depletion effects in mussel rafts of Galician Rias. Aquaculture. 2008;274(2–4):300–12. 10.1016/j.aquaculture.2007.11.025

[10] Hay CH. The dispersal of sporophytes of *Undaria pinnatifida* by coastal shipping in New Zealand, and implications for further dispersal of *Undaria* in France. Brit Phycol J. 1990;25(4):301–13. 10.1080/00071619000650331

[11] Mirto S, La Rosa T, Danovaro R, Mazzola A. Microbial and meiofaunal response to intensive mussel-farm biodeposition in coastal sediments of the Western Mediterranean. Mar Pollut Bull. 2000;40(3):244–52. 10.1016/S0025-326X(99)00209-X

[12] Dahlbäck B, Gunnarsson LÅH. Sedimentation and sulfate reduction under a mussel culture. Mar Biol. 1981;63(3):269–75. 10.1007/BF00395996

[13] Kaspar HF, Gillespie PA, Boyer IC, MacKenzie AL. Effects of mussel aquaculture on the nitrogen cycle and benthic communities in Kenepuru Sound, Marlborough Sounds, New Zealand. Mar Biol. 1985;85(2):127–36. 10.1007/BF00397431

[14] Wong KLC, O'Shea S. The effects of a mussel farm on benthic macrofaunal communities in Hauraki Gulf, New Zealand. N Z J Mar Freshwat Res. 2011;45(2):187–212. 10.1080/00288330.2010.550628

[15] Hartstein ND. Acoustical and sedimentological characterization of substrates in and around sheltered and open-ocean mussel aquaculture sites and its bearing on the dispersal of mussel debris. IEEE J Ocean Eng. 2005;30(1):85–94. 10.1109/JOE.2004.841388

[16] Castine SA, Bourne DG, Trott LA, McKinnon DA. Sediment microbial community analysis: Establishing impacts of aquaculture on a tropical mangrove ecosystem. Aquaculture. 2009;297(1–4):91–8. 10.1016/j.aquaculture.2009.09.013 25587201

[17] Giles H, Broekhuizen N, Bryan KR, Pilditch CA. Modelling the dispersal of biodeposits from mussel farms: The importance of simulating biodeposit erosion and decay. Aquaculture. 2009;291(3–4):168–78. 10.1016/j.aquaculture.2009.03.010

[18] Cranford PJ, Hargrave BT, Doucette LI. Benthic organic enrichment from suspended mussel (*Mytilus edulis*) culture in Prince Edward Island, Canada Aquaculture. 2009;292:189–96. 10.1016/j.aquaculture.2009.04.039

[19] Callier MD, McKindsey CW, Desrosiers G. Evaluation of indicators used to detect mussel farm influence on the benthos: Two case studies in the Magdalen Islands, Eastern Canada. Aquaculture. 2008;278:77–88. 10.1016/j.aquaculture.2008.03.026

[20] Carroll ML, Cochrane S, Fieler R, Velvin R, White P. Organic enrichment of sediments from salmon farming in Norway: Environmental factors, management practices, and monitoring techniques. Aquaculture. 2003;226(1–4):165–80. 10.1016/S0044-8486(03)00475-7

[21] Karakassis I, Tsapakis M, Smith CJ, Rumohr H. Fish farming impacts in the Mediterranean studied through sediment profiling imagery. Mar Ecol Prog Ser. 2002;227:125–33. 10.3354/meps227125

[22] Wildish DJ, Hargrave BT, MacLeod C, Crawford C. Detection of organic enrichment near finfish net-pens by sediment profile imaging at SCUBA-accessible depths. J Exp Mar Biol Ecol. 2003;285–286:403–13. 10.1016/S0022-0981(02)00540-3

[23] Diaz RJ, Schaffner LC. Comparison of sediment landscapes in the Chesapeake Bay as seen by surface and profile imaging In: Lynch MP, Krome EC, editors. Understanding the Estuary: Advances in Chesapeake Bay Research. 129. Baltimore, Maryland: Chesapeak Research Consortium; 1988 p. 222–40.

[24] Rhoads DC, Germano JD. Characterization of organism–sediment relations using sediment profile imaging: an efficient method of remote ecological monitoring of the seafloor (Remots system). Mar Ecol Prog Ser. 1982;8:115–28.

[25] Rhoads DC, Germano JD. Interpreting long-term changes in benthic community structure: a new protocol. Hydrobiologia. 1986;142(1):291–308. 10.1007/bf00026766

[26] Nilsson HC, Rosenberg R. Benthic habitat quality assessment of an oxygen stressed fjord by surface and sediment profile images. J Mar Syst. 1997;11(3–4):249–64. 10.1016/S0924-7963(96)00111-X

[27] O'Connor BDS, Costelloe J, Keegan BF, Rhoads DC. The use of REMOTS technology in monitoring coastal enrichment resulting from mariculture. Mar Pollut Bull. 1989;20(8):384–90. 10.1016/0025-326x(89)90316-0

[28] O'Reilly R, Kennedy R, Patterson A, Keegan BF. Ground truthing sediment profile imagery with traditional benthic survey data along an established disturbance gradient. J Mar Syst. 2006;62:189–203. 10.1016/j.jmarsys.2006.01.008

[29] Diaz RJ, Hansson LJ, Rosenberg R, Gapcynski PC, Unger MA. Rapid sedimentological and biological assessment of hydrocarbon contaminated sediments. Water, Air, Soil Pollut. 1993;66(3–4):251–66. 10.1007/BF00479849

[30] Bull DC, Williamson RB. Prediction of principal metal-binding solid phases in estuarine sediments from color image analysis. Environ Sci Technol. 2001;35:1658–62. 10.1021/es0015646 11329717

[31] Bagarinao T. Sulfide as an environmental factor and toxicant: Tolerance and adaptations in aquatic organisms. Aquat Toxicol. 1992;24(1–2):21–62. 10.1016/0166-445X(92)90015-F

[32] Thode-Andersen S, Jørgensen BB. Sulfate reduction and the formation of ^35^S-labeled FeS, FeS_2_, and S^0^ in coastal marine sediments. Limnol Oceanogr. 1989;34(5):793–806. 10.4319/lo.1989.34.5.0793

[33] Berner RA. Sulfate reduction and the rate of deposition of marine sediments. Earth Planet Sci Lett. 1978;37(3):492–8. 10.1016/0012-821X(78)90065-1

[34] Brüchert V. Early diagenesis of sulfur in estuarine sediments: The role of sedimentary humic and fulvic acids. Geochim Cosmochim Acta. 1998;62(9):1567–86. 10.1016/S0016-7037(98)00089-1

[35] Cornwell JC, Sampou PA. Environmental Controls on Iron Sulfide Mineral Formation in a Coastal Plain Estuary In: Viaravamurthy MA, Schoonen MAA, editors. Geochemical Transformations of Sedimentary Sulfur. ACS Symposium Series. Washington DC: ACS Symposium, 612; 1995 p. 224–42.

[36] Oenema O. Sulfate reduction in fine-grained sediments in the Eastern Scheldt, southwest Netherlands. Biogeochemistry. 1990;9(1):53–74. 10.1007/BF00002717

[37] Sorokin YI, Zakuskina OY. Acid-labile sulfides in shallow marine bottom sediments: A review of the impact on ecosystems in the Azov Sea, the NE Black Sea shelf and NW Adriatic lagoons. Estuar Coast Shelf Sci. 2012;98:42–8. 10.1016/j.ecss.2011.11.020

[38] Wilson PS, Vopel K. Estimating the in situ distribution of acid volatile sulfides from sediment profile images. Limnol Oceanogr Methods. 2012;10:1070–7. 10.4319/lom.2012.10.1070

[39] Muggeo VMR. Estimating regression models with unknown break-points. Stat Med. 2003;22:3055–71. 10.1002/sim.1545 12973787

[40] R Core Team. R: A language and environment for statistical computing. R Foundation for Statistical Computing, Vienna, Austria URL http://www.R-project.org. 2013.

[41] Mackay KA, Mackay EJ, Neil HL, Mitchell JS, Bardsley SA, cartographers. Hauraki Gulf. NIWA Chart, Miscellaneous Series 91: National Institute of Water & Atmospheric Research Ltd.; 2012.

[42] Chamberlain J, Fernandes TF, Read P, Nickell TD, Davies IM. Impacts of biodeposits from suspended mussel (*Mytilus edulis* L.) culture on the surrounding surficial sediments. ICES J Mar Sci. 2001;58:411–6. 10.1006/jmsc.2000.1037

[43] Giles H, Pilditch CA. Effects of diet on sinking rates and erosion thresholds of mussel *Perna canaliculus* biodeposits. Mar Ecol Prog Ser. 2004;282:205–19. 10.3354/Meps282205

[44] Hartstein ND, Stevens CL. Deposition beneath long-line mussel farms. Aquacult Eng. 2005;33(3):192–213. 10.1016/j.aquaeng.2005.01.002

[45] Callier MD, Richard M, McKindsey CW, Archambault P, Desrosiers G. Responses of benthic macrofauna and biogeochemical fluxes to various levels of mussel biodeposition: An in situ "benthocosm" experiment. Mar Pollut Bull. 2009;58(10):1544–53. 10.1016/j.marpolbul.2009.05.010 19541330

[46] McKindsey CW, Archambault P, Callier MD, Olivier F. Influence of suspended and off-bottom mussel culture on the sea bottom and benthic habitats: a review. Canadian Journal of Zoology. 2011;89(7):622–46. 10.1139/z11-037

[47] Robert P, McKindsey CW, Chaillou G, Archambault P. Dose-dependent response of a benthic system to biodeposition from suspended blue mussel (*Mytilus edulis*) culture. Mar Pollut Bull. 2013;66(1–2):92–104. 10.1016/j.marpolbul.2012.11.003 23219398

