# Supplementary material for: Assessing the Sulfide Footprint of Mussel Farms with Sediment Profile Imagery: A New Zealand Trial
Source: PLoS One. 2015 Jun 17;10(6):e0129894. doi: 10.1371/journal.pone.0129894 (PMC4471228; doi:10.1371/journal.pone.0129894)
Supplement: S1 Table — Measured sediment color intensities along three transects running across the boundary of the mussel farm in Awakiriapa Bay, Waiheke Island, New Zealand were extrapolated over an area extending from ~50 m inside the northern fam boundary to ~200 m north of the farm boundary. (DOCX) [file pone.0129894.s001.docx]

S1 Table. Parameters for the local polynomial interpolation model in ArcMap (ESRI ArcGIS, version 10.2). Measured sediment color intensities along three transects running across the boundary of the mussel farm in Awakiriapa Bay, Waiheke Island, New Zealand were extrapolated over an area extending from ~50 m inside the northern fam boundary to ~200 m north of the farm boundary.

| Parameter | Setting or value |
| --- | --- |
| method | local polynomial interpolation |
| kernel function | exponential |
| output type | prediction |
| power | 1 |
| bandwidth | 166.62 |
| spatial condition number threshold | 30 |
| exploratory trend surface analysis | 20 |
| searching neighborhood | standard |
| neighbors to include | 1000 |
| include at least | 10 |
| sector type | full |
| major semiaxis | 208.27 |
| minor semiaxis | 208.27 |
| angle | 0 |
| cell size | 1.2 m |
